# Supplementary material for: The Construction and Exploration of a Comprehensive MicroRNA Centered Regulatory Network in Foxtail Millet (Setaria italica L.)
Source: Front Plant Sci. 2022 May 6;13:848474. doi: 10.3389/fpls.2022.848474 (PMC9121102; doi:10.3389/fpls.2022.848474)
Supplement: Supplementary file 6 [file Data_Sheet_6.zip › Sit-miRNovel2_Seita.5G215500.1_1080_TPlot.pdf]

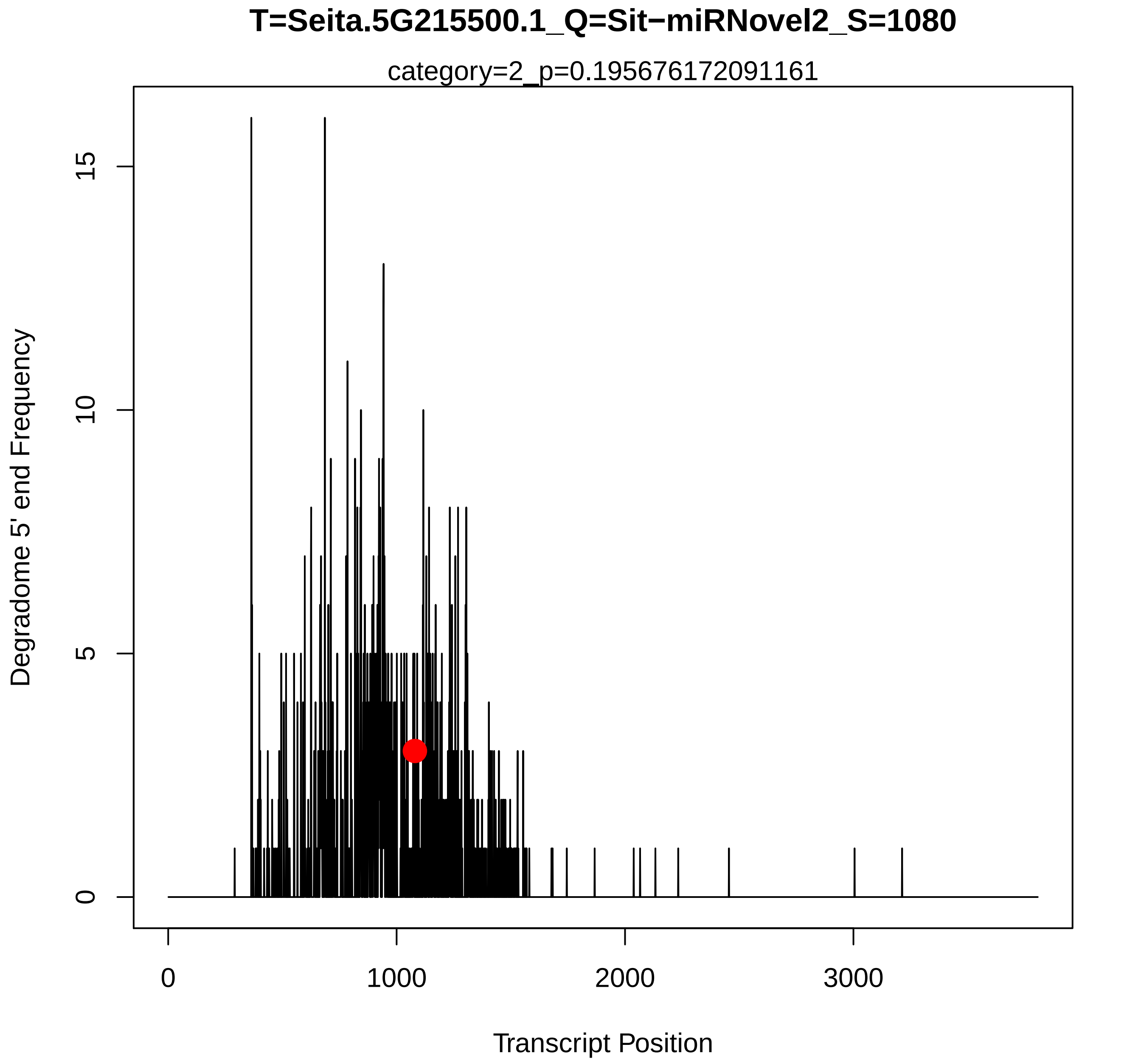

T=Seita.5G215500.1_Q=Sit−miRNovel2_S=1080
category=2_p=0.195676172091161
Degradome 5' end Frequency
0
5
10
15
0
1000
2000
3000
Transcript Position
